# Compensating for cross-reactions using avidity and computation in a suspension multiplex immunoassay for serotyping of Zika versus other flavivirus infections

**DOI:** 10.1007/s00430-017-0517-y

**Published:** 2017-08-29

**Authors:** Bengt Rönnberg, Åke Gustafsson, Olli Vapalahti, Petra Emmerich, Åke Lundkvist, Jonas Schmidt-Chanasit, Jonas Blomberg

**Affiliations:** 1 Section of Clinical Microbiology, Department of Medical Sciences, Uppsala Academic Hospital, Uppsala University, 751 85 Uppsala, Sweden; 2 Department of Medical Biochemistry and Microbiology, Zoonosis Science Center, Uppsala University, Uppsala, Sweden; 3 Laboratory of Clinical Microbiology, Uppsala University Hospital, Uppsala, Sweden; 4 Department of Veterinary Biosciences and Virology, University of Helsinki and Helsinki University Hospital, Helsinki, Finland; 5 WHO Collaborating Centre for Arbovirus and Haemorrhagic Fever Reference and Research, Bernhard Nocht Institute for Tropical Medicine, 20359 Hamburg, Germany; 6 Department of Tropical Medicine and Infectious Diseases, Center of Internal Medicine II, University of Rostock, 18057 Rostock, Germany; 7 German Centre for Infection Research (DZIF), Partner Site Hamburg-Luebeck-Borstel, Hamburg, Germany

**Keywords:** Zika virus, Dengue virus, Flavivirus, Suspension multiplex immunoassay, Serological cross-reaction, Pathogen surveillance

## Abstract

The recent spread of Zika virus (ZIKV) in the Americas and Asia necessitates an increased preparedness for improved maternal and perinatal health and blood safety. However, serological cross-reactions, especially to Dengue virus (DENV), complicate ZIKV antibody serodiagnosis. A novel “pan-Flavi” suspension multiplex immunoassay (PFSMIA) using 25 antigens, whole virus (WV), non-structural protein 1 (NS1), and envelope (E) proteins, from 7 zoonotic flaviviruses for specific detection of ZIKV and DENV IgM and IgG was developed. Patterns of antibody cross-reactivity, avidity, and kinetics were established in 104 sera from returning travelers with known ZIKV and DENV infections. PFSMIA gave IgM- and IgG-sensitivities for both viruses of 96–100%, compared to an immunofluorescence assay. Main IgM cross-reactions were to NS1, for IgG to the E and WV antigens. Infecting virus yielded reactivity to several antigens of the homologous virus, while cross-reactions tended to occur only to a single antigen from heterologous virus(es). A specificity-enhancing computer procedure took into account antibody isotype, number of antibody-reactive antigens per virus, avidity, average degree of cross-reactivity to heterologous flavivirus antigens, and reactivity changes in serial sera. It classified all 50 cases correctly. Applied to sera from 200 pregnant women and 173 blood donors from Sweden, one blood donor was found ZIKV NS1 IgM positive, and another as ZIKV NS1 IgG positive. These samples did not react with other ZIKV antigens and were thereby judged as false-positives. PFSMIA provided sensitive and specific ZIKV and DENV serology, warranting high-throughput serological surveillance and a minimized need for laborious and expensive virus neutralization assays.

**Electronic supplementary material:**

The online version of this article (doi:10.1007/s00430-017-0517-y) contains supplementary material, which is available to authorized users.

## Introduction

Zika virus (ZIKV) infections emerged during the last decade in several parts of the world, most dramatically during 2016, and are now affecting millions [1]. Its global distribution is, however, not yet well understood. ZIKV infection during pregnancy is linked to severe congenital disorders. This will necessitate extensive surveillance of pregnant women and calls for reliable methods with high-throughput [2]. Flavivirus serology is notoriously difficult due to antigenic cross-reactions within the genus [3–9], which requires simultaneous testing of antibodies to several flaviviruses. Testing by several monoplex assays is slow, expensive, and labor intensive. The “gold standard” virus neutralization test is cumbersome, requires strict biosafety, and has additional limitations [4]. We designed a “pan-Flavi” suspension multiplex immunoassay (PFSMIA) [10–13] covering ten of the most medically important flaviviruses, for comparative purposes also the clinically similar chikungunya virus (CHIKV), using several antigens for most viruses. In this paper, we focus on ZIKV and DENV. The observed cross-reactions were antigen and immunoglobulin (Ig)-type specific. We explored two ways of enhancing ZIKV serology. First, we developed a data reduction procedure that used the average degree of cross-reactivity and completeness (coincidence criterion) of homologous antibody responses, similar to the principle of an immunoblot confirmation test for HIV. It could partially compensate for heterologous, cross-reactive, signals. Second, we developed a rational avidity-based test. Re-incubating the beads from a previous PFSMIA run with urea removed nearly all initially observed cross-reactions, for both IgM and IgG of ZIKV and DENV cases. An automated diagnostic support procedure which weighed isotype, coincidence, avidity, and cross-reactivity pattern as well as development of reactivity in serial samples from the same person, was developed. We validated the method by sera from returning travelers with Zika Virus Disease (ZVD, *n* = 44) and dengue fever (DF, *n* = 60), blood donors (*n* = 173), and pregnant women (*n* = 200). The evaluation was focused on differentiating ZVD from DF. All of these known flavivirus infections were correctly classified in spite of cross-reactions to heterologous flavivirus(es). Two blood donor sera reacted to only one ZIKV antigen, and thereby judged as false positive. We demonstrated that taking antigen coincidence, average cross-reactivity, isotype, serial sampling, and avidity into account considerably increases the discrimination of ZVD from DF, and most likely, the specificity of flavivirus serology as a whole.

## Materials and methods

### Sera

There were 13 ZVD cases, travelers returning to Europe who acquired the ZIKV infection in Brazil, Colombia, Mesoamerica, Haiti, Antilles, or Martinique, and were diagnosed by ZIKV-specific RT-PCR, EIA, and indirect immunofluorescence test (IIFT) as previously described [14]. The 37 DF cases were returning travelers infected in either Sri Lanka, India, Thailand, Malaysia, Myanmar, Bali, Curacao, Philippines, Brazil, Bolivia, or Costa Rica. They were diagnosed by DENV-specific RT- PCRs, NS1 antigen assay, and IIFT.

We anonymously screened 200 sera from pregnant women collected during 2015 at the maternity ward of the Uppsala Academic Hospital for viral screening and from 173 blood donors collected during 2016 for blood bank virus screening, all obtained under the Swedish Biobank law, which includes donor consent.

Flavivirus antibody reference sera (*n* = 9), from WHO and elsewhere, were used for calibration of the classification support algorithms.

### Pan-Flavi suspension multiplex immunoassay (PFSMIA)

PFSMIA was mainly performed as previously described [11–13, 15]. Briefly, 5–10 µg of antigen was coupled to 200 µl (2.5 million) carboxylated differentially color-marked magnetic microspheres (MagPlex-C microspheres, Luminex Corp, TX, USA) using carbodiimide. After the coupling of antigen with beads, beads were incubated with 0.5 mL of PBS containing 0.05% (v/v) Tween 20 and 50 mM Tris (PBST) and subsequently washed with 0.5 mL StabilGuard (SurModics, Eden Prairie, MN, USA, #SG01-1000) using a magnetic separator (Life technologies #A14179). The bead pellet was finally resuspended in 400 µl StabilGuard. This created a bead mixture consisting of 6250 beads/µl.

The selection of antigens included both broadly reactive whole virus antigens (WV), recombinant glycosylated E proteins (E), and non-structural protein 1 (NS1) with the intention to cover the major zoonotic flaviviruses. The panel contained commercially available antigens of high purity and of as high antigenicity as possible. The low demand for amount of antigen in SMIA [11] enabled us to analyze a large number of sera using a limited amount of antigen. The following antigens were included: CHIKV baculovirus recombinant glycosylated E1 (wild type and mutated-A226 V; Aalto Bio Reagents, Dublin, Ireland, ProSpec Bio, Ness Ziona, Israel; included for monitoring the degree of false positivity to a non-flavi- but clinically confounding virus) and WV (ZeptoMetrix, Buffalo, NY, USA); DENV 1-4 NS1 (Native Antigen Company, Heyford Park, UK, who provided all NS1 glycoproteins in the study, produced in mammalian cells*) and WV (Microbix Inc, Mississauga, Ontario, Canada). DENV WV and NS1 reactions summed as DENVXWV and DENVXNS1, respectively; ZIKV; WV (ZeptoMetrix, Buffalo, NY, USA), NS1, baculo recombinant E (E; Aalto [ENV A] and Meridian Life science, Inc. Memphis, Tennessee, USA)[ENV M], yellow fever virus (YFV) NS1 and WV (ZeptoMetrix); tick-borne encephalitis virus (TBEV) WV (Jena Bioscience, Jena, Germany) and NS1; West Nile virus (WNV) NS1, E coli recombinant E (Jena*), WV (East Coast Bio, North Berwick, Maine, USA and ZeptoMetrix); Japanese Encephalitis Virus (JEV) NS1,* E. coli* recombinant E (Prospec*); Usutu virus (USUV) NS1. Antigens marked with an asterisk were His-tagged. Their degree of coupling to magnetic beads was monitored using an anti-His serum (Antibodies on line, ABIN100493) [11]. For all antigens except USUV, coupling was also monitored using known positive reference sera (Table S2, Supplementary material). USUV positive reference sera were not available.

For IgG determination 50 µl of serum diluted 1/50 in PBS (phosphate buffered saline), pH 7.4, containing 0.05% (v/v) Tween 20, 50 mM Tris, and 2% (v/v) Prionex (Sigma-Aldrich #81662) (PBSTP) was added to wells of a round bottom 96-well microtiter plate (Greiner #104650). Fifty µl of a vortexed and sonicated bead mixture consisting of 25 beads/µl suspended in PBSTP was then added to each well, giving a final serum dilution of 1/100, followed by 1 h incubation, washing with PBS using the magnetic plate separator. Subsequently, the beads were resuspended in 50 µl of PBSTP and 50 µl of biotinylated-protein G (Pierce, Article No. 29988) at the concentration of 4 µg/mL in PBSTP in each well, incubated for 30 min at room temperature (RT), washed PBS and resuspended in 50 µl of PBSTP, followed by the addition of fifty µl of streptavidin–phycoerythrin (SA-PhE) (InVitrogen, Thermo Fisher, article nr S-866) at the concentration of 4 µg/mL in PBSTP in each well and finally incubated for 15 min at RT. The beads were washed once with PBS before they were resuspended in 100 µl of PBS and analyzed in a Luminex-200 (Luminex Corporation, Austin, Texas, USA) instrument according to the instruction from the manufacturer. To detect any antibody binding to the beads themselves, a naked non-antigen-containing (“blank”) bead was included. One negative (“non-template”) control, where PBSTP instead of serum was added, was also used in all experiments. Positive control sera were also included to govern the repeatability of antibody reactivity levels.

For IgM determination, 1 μL of serum was preincubated with 9 μL of GullSORB (Meridian, nr XX715; to remove IgG which can compete and cause false positivity due to rheumatoid factors) for 10 min at RT, then 40 μL PBSTP was added. After that, each well was subjected to incubation with 50 μL bead mixture, followed by incubation with biotinylated anti-IgM (Sigma B1265) at the concentration of 4 µg/mL in PBSTP and streptavidin–phycoerythrin conjugate, as described above for detection of IgG.

The avidity selection started with microtiter wells that had been subjected to a previous PFSMIA and measured by the Luminex 200 flow meter, thus containing magnetic color-coded beads with antigen–antibody complexes plus signal-generating molecules. They were washed with 100 µL of PBS and then incubated with 100 µL of 8 M urea for 1 h at RT. Finally, the excess of the urea solutions was removed, and the well washed with 100 µL of PBS. After this step, each well was subjected to incubation with biotinylated anti-immunoglobulin, followed by streptavidin conjugated with phycoerythrin, as described above for the normal PFSMIA procedure. An avidity index (AI) was calculated as the ratio between MFI after and before urea treatment, respectively.

The PFSMIA and its support algorithms were preliminarily calibrated using sera from Swedish, Colombian, and Mozambican patients with CHIKV, TBEV, ZIKV, and DENV infections, WHO reference sera for YFV, TBEV, and WNV (Table S2, Supplementary material), as well as sera from healthy Swedish blood donors. The cutoff was calculated as the average Median Fluorescence Intensity (MFI) + 3 standard deviations; based on TBEV antibody negative Swedish blood donor sera. Intra-assay variation was below 11% [11], justifying the twofold significance limit for reactivity change used here. If not otherwise indicated, all sera were tested at a final dilution of 1/100.

### Diagnostic support procedure

Briefly, the algorithm (written in Visual FoxPro by JB [16]) favors coincident reactions on several antigens from a virus, like in a Western blot, producing a score (program “Flaviclass,” Table 1). The tendency for cross-reaction of a virus to other flaviviruses was calculated as average ratios between heterologous, recipient, antigen (“To”), and homologous, donor, virus (“From”) MFI, for both IgG and IgM, with and without urea treatment. The ratios were used to create a “Final score,” with an arbitrary cutoff of 1. Avidity indices (see below) were calculated if possible. Average cross-reactivity factors were calculated from data of the 50 cases of this study. The cross-reactivity factors were similar to those calculated from a larger study using Swedish, Colombian, and Mozambican cases of ZIKV, DENV, and TBEV infections (to be published), and from purchased reference sera derived from WNV-, JEV-, and YFV-infected individuals (Table S2. Supplementary material). The algorithm infers “TBEV infection” if antibodies to both WV and NS1 are present, “TBEV vaccination” if only WV antibodies are present after compensation for expected cross-reactivity. If the TBE WV antibodies are of low avidity (AI of 0.4 and lower), and a DENV infection (presence of both WV and NS1 antibodies) is detected, a TBEV cross-reaction from DENV is assumed. This will be addressed in a forthcoming article.

**Table 1:** Steps in the computer program “Flaviclass”

Step	Action
1	Quality control, quantitative vs reference sera; checking timing of sera
2	Summing DENV 1–4 type reactions to “DENVx,” subtraction of antigen-specific cutoffs
3	Calculation of avidity indices
4	Calculation of coincidence factors
5	Estimation of cross-reactivity, based on x factors and avidity
6	Calculation of final score, per flavivirus, per serum
7	Detection of more than twofold reactivity change in serial samples
8	Judgement of patient; primary or previous infection

The evidence for an infection of a serum with a certain flavivirus is quantified as two scores (Table 1 steps 3–6): 1. a positive score based on MFI of antigens from the virus weighted to favor coincident reactions, and 2. a negative score embodying expected cross-reactions based on cross-reactivity factors and avidity. A final score (positive score–negative score) was then calculated for each serum, antibody isotype, and presence or absence of urea (step 6 of Table 1).

In case several serial samples from the same case were present, the algorithm automatically looked for activity changes (at least twofold; step 7 of Table 1), and suggested a likely judgement regarding the patient case, including “Primary” and/or “Previous” flavivirus infection encountered by the patient (step 8 of Table 1). The choice of a twofold difference of MFI as criterion for significant antibody change was based on the high reproducibility of SMIA [11], and our previous experience of single dilution serology [17]. Step 8 weighs the information on isotype and reactivity change into a diagnostic score for primary and previous infection, respectively.

### Avidity selection of anti-flavivirus IgM and IgG

The avidity selection started with microtiter wells that had been subjected to a previous PFSMIA and measured by the Luminex 200 flow meter, thus containing magnetic color-coded beads with antigen–antibody complexes plus signal-generating molecules. They were re-measured after urea treatment and reincubation with signal-generating molecules.

## Results

### Calculation of cutoffs

Cutoffs were calculated based on the results from seronegative Swedish (Uppsala) blood donor sera, a population with a low incidence of ZIKV and DENV infections. Thus, we took advantage of the relatively low flavivirus burden of the Northern European population for establishing flavivirus cross-reactivities in a previously flavivirus naïve patient. For the purpose of defining “seronegativity,” we had to detail the exclusion criteria. Among the 173 blood donor sera, previous TBE vaccination was inferred as the presence of only a singular (MFI > 500) TBEV WV IgG signal (*n* = 42), previous YFV vaccination as the presence of only a strong YFV NS1 IgG (MFI > 500, *n* = 4). Two blood donor sera reacted strongly with IgG to both YFV NS1 and TBEV but no other flavivirus antigens, and were judged as probable YFV and TBEV vaccinees. Seven reacted strongly with both TBEV WV and TBEV NS1, judged as previous TBEV infections. Although this is not a TBEV epidemiological study, both asymptomatic and symptomatic TBEV infection is relatively common in the Uppsala area, with at least 15 cases/year/100,000 persons [18]. The observed TBE WV and NS1 antibody frequency (7/173 blood donors and 7/200 mothers) was thus somewhat higher than expected (1.7/373). Cutoffs were calculated from the results of 45 of the remaining “seronegative” flavivirus antibody blood donor sera. As shown in Table S1 (see Supplementary material), most antigens gave low cutoffs. WV antigens and ZIKV E cutoffs were higher. Subtraction of cutoff is step 2 of the diagnostic support procedure (Table 1).

### Sensitivity and specificity of DENV and ZIKV antibody detection

The serum-specific background, of the “naked bead,” was on average, for IgG; 50 MFI; *n* = 45 and for IgM 30 MFI; *n* = 46. Those values were first subtracted for each serum. Then, an antigen-specific cutoff based on the average of 45 DENV, TBEV, and YFV seronegative Swedish Blood donors, plus 3 times standard deviation, (Table S1, see Supplementary material) was subtracted. Only one of 173 blood donors and none of 200 pregnant women reacted for IgG, and one for IgM, with ZIKV NS1 (section “Time course of homologous and heterologous (cross-reactive) flavivirus antibodies during ZIKV and DENV infection”), indicating a high specificity of PFSMIA for ZIKV and DENV IgG and IgM. A sensitivity of 96–100% vs indirect immunofluorescence (IIFT) was observed (Table 2).

**Table 2:** Fourfold tables, ZIKV and DENV, IgM and IgG for SMIA versus indirect immunofluorescent test, IIFT

ZIKV IgM SMIA step 2^a^	ZIKV IgM IIFT		
	Pos	Neg	
Pos	26	5	
Neg	1	9	
			Sensitivity
		Total 41	ZIKV SMIA IgM vs IIF IgM 26/27 = 96%

ZIKV IgG SMIA step 2^a^	ZIKV IgG IIFT		
	Pos	Neg	
Pos	39	2	
Neg	0	0	
			Sensitivity
		Total 41	ZIKV SMIA IgG vs IIF IgG 39/39 = 100%

DENV IgM SMIA step 2^a^	DENV IgM IIFT		
	Pos	Neg	
Pos	43	13	
Neg	1	1	
			Sensitivity
		Total 8	DENV SMIA IgM vs IIF IgM 43/44 = 98%

DENV IgG SMIA step 2^a^	DENV IgG IIFT		
	Pos	Neg	
Pos	58	0	
Neg	2	0	
			Sensitivity
		Total 60	DENV SMIA IgG vs IIF IgG 60 = 97%

^a^Step 2, Table 1. ZIKV SMIA result based on NS1, DENV SMIA result based on WV and NS1 antigens

With regard to clinical sensitivity, the PFSMIA and the diagnostic support procedure yielded a “Primary ZIKV” antibody classification for 12 of 13 ZVD cases, a “Previous ZIKV” for 1 of 13 ZVD cases, and a “Primary DENV” antibody classification for all 37 DF cases (section “Time course of homologous and heterologous (cross-reactive) flavivirus antibodies during ZIKV and DENV infection”) (Table 2). A larger cohort, with a greater diversity of flavivirus infections, is needed for a future more critical examination of sensitivity and specificity.

### Overview of patients and samples

Table 3 gives basic information for the 13 ZVD and 37 DF cases, as well as the IgM and IgG outcomes per sample (“Main serological reactions and activity changes”) and final outcome per case (“Case judgement”). The latter two will be further discussed at the end of the “Results” section. An imperfect correlation between DENV type and the maximally reactive DENV type WV and NS1 antigen was observed, see Supplementary material.

**Table 3:** Oe of PFSMIA

Case	Travel	Known vaccinations	Days post first symptom of sera	Previous diagnostic evidence	Dengue type^a^	Main serological reactions (with activity changes), PFSMIA (steps 6 and 7)^b^	Case judgement, PFSMIA (step 8)^c^	Concordance
ZIKV disease								
Z1	Brazil, Sao Paulo		20,37,249	IgM		M: ZIKV (zik §3/1 0.00). G: ZIKV, TBEV_vacc (zik !2/1 1.57)	Primary: ZIKV, Previous: DENV?, TBEVACC?	OK
Z2	Brazil Sao, Paulo		20,37.249	IgM		M: ZIKV? (zik §2/1 0.23) G: TBEV_vacc?	Primary: ZIKV Previous: DENV	OK
Z3	Colombia		10,15,66,207,233	IgM		M: ZIKV (zik §4/1 0.01), G: (zik !4/1 12)	Primary: ZIKV	OK
Z4	Martinique		11,16,36	IgM		M: ZIKV (zik !2/1 4.0, zik §3/2 0.48), G: (zik !3/1 > 100)	Primary: ZIKV	OK
Z5	Martinique		2,7,27	IgM		M: (zik !2/1 21, zik §3/2 0.37), G: (zik !3/1 > 100)	Primary: ZIKV	OK
Z6	Mesoamerica	TBEV	114	PCR/s, PCR/u, IgM, etc.		G: ZIKV	Previous: ZIKV	OK
Z51	Colombia		66,119,176,211,289, 309, 365, 408	IgM, NT		M: ZIKV? (zik §6/1 0.00), G: ZIKV (zik §8/2 0.50)	Primary: ZIKV	OK
Z52	Brazil, Sao Paolo	TBEV 1994	39,248,435	IgM		M: ZIKV? (zik §3/1 0.06), G: ZIKV, TBEV_vacc (zik §3/1 0.39, tbevacc §3/1 0.36)	Primary: ZIKV, Previous: TBEVACC?	OK
Z53	Brazil, Sao Paolo	TBEV 1994	403,246, 427	IgM		M: ZIKV, G: ZIKV, TBEV_vacc, (tbevacc §3/1 0.49)	Primary: ZIKV, Previous: TBEVACC?	OK
Z54	Martinique		12,17,38,53,116	PCR/u, IgM		M: ZIKV, YFV? (zik §4/1 0.04, yfv §2/1 0.04) G: (zik !3/1 5.8)	Primary: ZIKV, YFV	OK
Z55	Martinique		3,8,28,45,101	PCR/u, IgM, NT		M: ZIKV (zik §4/1 0.00), G: (zik !4/1 7.6)	Primary: ZIKV, DENV?	OK
Z56	Haiti, Porte au Prince	YFV 2011, TBEV 2003	20	PCR/u, NT		M: ZIKV, G: ZIKV, TBEV_vacc, YFV?	Primary: ZIKV, Previous: TBEVACC	OK
Z57	The Netherlands, ABC islands	YFV	1,8,23,56	PCR/u, IgM		M: ZIKV, YFV? (zik §3/1 0.19). G: ZIKV, YFV? (zik !2/1 1.94, yfv §3/2 0.35)	Primary: ZIKV, TBEVACC?	OK
DENV fever								
D1	Myanmar		6,42	Ag	DENV2	M: (den !2/1 5.3)	Primary: DENV	OK
D2	Thailand		14,32	Ag	DENV3	M: (den !2/1 > 100, yfv !2/1 > 100), G: TBEV_vacc? (den !2/1 11, yfv !2/1> 100, tbevacc !2/1 2.3)	Primary: YFV, DENV	OK?
D3	Thailand		14,32	Ag	DENV1	G: (den !2/1 8.2)	Primary: DENV	OK
D30	Thailand		11	Ag	DENV3	M: DENV	Primary: DENV	OK
D31	Thailand		9	Ag	DENV1	M: DENV G: TBEV_vacc?	Primary: DENV	OK
D32	Thailand		40	Ag	DENV1	M: DENV G: TBEV_vacc?	Primary: DENV	OK
D35	Curacao		35	Ag	DENV3	M: DENV G: DENV?, TBEV_vacc?	Primary: DENV	OK
D38	Thailand		47	Ag	DENV4	M: DENV G: DENV, TBEV_vacc?	Primary: DENV	OK
D39	Philippines		U	Ag	DENV1	M: DENV G: TBEV_vacc	Primary: DENV, Previous: TBEVACC?	OK
D40	Thailand		U	Ag	DENV1	M: DENV, TBEV_vacc? G: DENV	Primary: DENV	OK
D43	Thailand		13	Ag	DENV1	M: DENV	Primary: DENV	OK
D45	Bolivia		14	Ag	DENV4	M: DENV, TBEV_vacc? G: TBEV_vacc?	Primary: DENV, TBEVACC?	OK
D46	Costa Rica		U	Ag	DENV2	M: DENV G: TBEV_vacc?	Primary: DENV	OK
D51	Sri Lanka		4,18,44,50,206,244,339	PCR/s, IgM, Ag	DENV1	M: DENV(den !2/1 4.9,den §5/2 0.13) G: DENV (den !3/1 38, den §6/3 0.41)	Primary: DENV	OK
D52	Bali		14	PCR/s, Ag	DENV1	M: DENV G: TBEV_vacc	Primary: DENV, Previous: TBEVACC	OK
D53	India, New Delhi		14	PCR/s, IgM	DENV1	M: DENV G: DENV, TBEV_vacc?	Primary: DENV	OK
D54	U		U	IgM	DENV1	M: DENV, G: DENV	Primary: DENV	OK
D55	Thailand		10,440	IgM	DENV1	M: DENV, TBEV_vacc? (den §3/1 0.22, yfv !2/1 4.4, tbevacc §2/1 0.36 .00) G: TBEV_vacc (den !3/2 3.7)	Primary: DENV, TBEVACC, YFV?	OK
D56	Thailand		21,262,634,	PCR/s, IgM	DENV2	M: DENV, ZIKV (den §3/1 0.19) G: DENV (den !2/1 3.0)	Primary: DENV, ZIKV?	OK?
D57	Brazil		10,40	IgM	DENV2	M: DENV, G: DENV, YFV?, USUV? (usu §2/1 0.40)	Primary: DENV, Previous: YFV?	OK
D58	Thailand		U	IgM	DENV2	M: DENV (den §3/1 0.11 .00) G: DENV, TBEV_vacc (den !2/1 2.6)	Primary: DENV	OK
D59	U		U	Ag	DENV2	G: DENV, TBEV_vacc? (den §3/1 0.34, tbevacc §3/1 0.34)	Previous: DENV, TBEVACC?	OK
D60	U		U	IgM	DENV2	G: DENV	Previous: DENV	OK
D61	Thailand		U	Ag, IgM	DENV3	M: DENV (den §3/2 0.30) G: (den !3/1 33)	Primary: DENV	OK
D62	Malaysia, Bali	TBEV as child	1,5,19,52	PCR/s, Ag, IgM	DENV3	M: YFV?, DENV G: TBEV_inf (den !3/1 3.9)	Primary: DENV, YFV?, Previous: TBEINF	OK?
D63	U		4	PCR/s, Ag, IgM	DENV4	M: DENV	Primary: DENV	OK
D64	U		6	PCR/s, Ag, IgM	DENV2	M: DENV G: TBEV_vacc	Primary: DENV, Previous: TBEVACC?	OK
D65	U		7	PCR/s, Ag, IgM	DENV2	M: DENV G: TBEV_vacc	Primary: DENV, Previous: TBEVACC?	OK
D66	U		16	PCR/s, Ag	DENV4	M: DENV G: DENV, USUV?	Primary: DENV	OK
D67	U		6	PCR/s, Ag, IgM	DENV3	M: DENV	Primary: DENV	OK
D68	U		4	PCR/s, Ag, IgM	DENV4	M: DENV, ZIKV G: TBEV_vacc	Primary: DENV, ZIKV?, Previous: TBEVACC	OK
D69	U		11	PCR/s, Ag, IgM	DENV2	M: DENV? G: DENV	Primary: DENV	OK
D70	U		8	PCR/s, Ag, IgM	DENV1	M: DENV G: DENV	Primary: DENV	OK
D71	U		12	PCR/s, Ag, IgM	DENV2	M: DENV, G: TBEV_vacc	Primary: DENV, Previous: TBEVACC	OK
D72	U		U	PCR/s, Ag, IgM	DENV3.4	M: DENV, G: TBEV_vacc, DENV	Primary: DENV, Previous: TBEVACC	OK
D73	U		8	PCR/s, Ag, IgM	DENV1	M: DENV G: TBEV_vacc, DENV?	Primary: DENV, Previous: TBEVACC	OK
D74	U		12	PCR/s, Ag, IgM	DENV2	M: DENV G: TBEV_vacc?	Primary: DENV	OK

*PCR/u* PCR in urine, *PCR/s* PCR in serum, *Ag* DENV antigen detection, *NT* neutralization test, *U* unknown

^a^Samples from patient D72 were positive for both DENV3 and DENV4

^b^This column summarizes all reactions of sera belonging to the patient at program steps 6 and 7 of Table 1. *M* IgM, *G* IgG. Activity changes are within brackets. ! = rise, § = decline, followed by High Serum nr/Low Serum nr and ratio. Abbreviations: zik = ZIKV, Zika, den = DENV, Dengue, usu = USUV, Usutu, TBE_vacc, tbevacc = TBE vaccination, TBE_inf = TBE infection

^c^This column contains results of program step 8 of Table 1

### Flavivirus antibody patterns during ZIKV infection

**Fig. 1:**
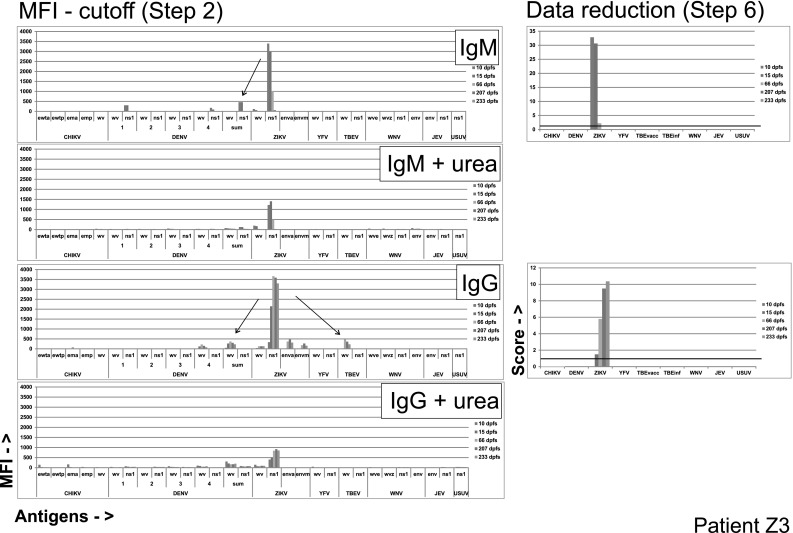
Evolution of anti-flavivirus antibodies in a ZVD case (Z3) with few cross-reactions. Original data after cutoff subtraction (*left panels*, corresponding to step 2 of Table 1) and processed final scores (“Data reduction,” with a score cutoff of 1, *right panels*, corresponding to step 6 of Table 1), for IgM and IgG, with or without urea treatment, are shown. Results from sera taken 10–233 days post first symptom (dpfs) are shown). Probable cross-reactions are shown as *arrows*. *MFI* Median Fluorescence Intensity. *X*-axis antigens are whole virus (“WV”), NS1 or recombinant envelope (“env” or “E”), CHIKV antigens are recombinant E1 protein, wild type (“wt”) and mutated (“m”) from two different manufacturers. DENV antigens are WV and NS1 for each serotype, and summed. ZIKV antigens included recombinant E from two different manufacturers. WNV antigens included WV from two manufacturers (See “Materials and methods”)

**Fig. 2:**
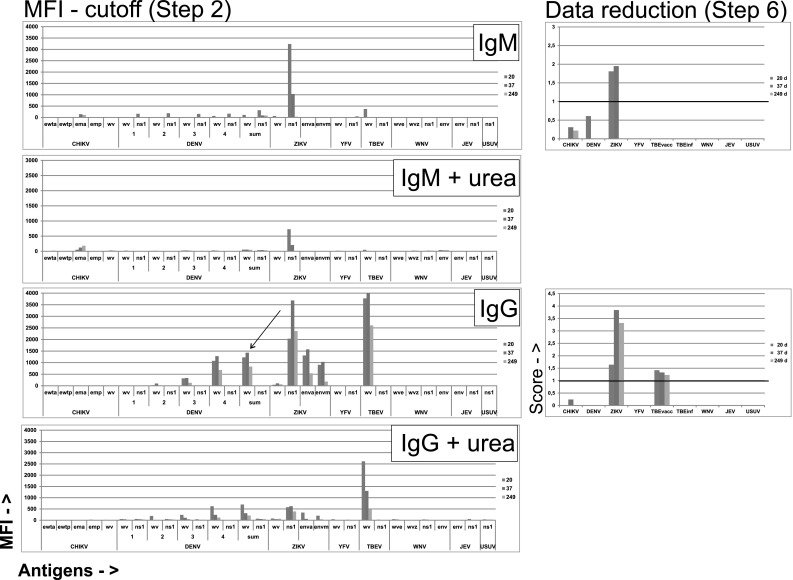
Evolution of anti-flavivirus antibodies in a ZVD case (Z1) with a more complex serological pattern. Original data after cutoff subtraction (*left column*) and processed final scores (*right column*), for IgM and IgG, with or without urea treatment, respectively, are shown. For further explanation, see legend of Fig. 1

The sera from all 13 ZVD cases displayed a PFSMIA IgG and/or IgM pattern dominated by a ZIKV non-structural protein 1 (NS1) reaction, accompanied by either E or WV reactions or both. All 13 sera also showed more or less pronounced DENV cross-reactions, mainly to NS1 for IgM, and to WV for IgG. SMIA results of two ZVD cases, one with little and one with more serological complication, are shown in Figs. 1 and 2, respectively.

Case Z3 was a male returning to Germany from Colombia. ZIKV IgM was detected in serum by IIFT on day 10. Figure 1 shows an almost exclusive anti-ZIKV IgM response, peaking at day 10, with a slight cross to DENV NS1, and a subsequent almost as specific anti-ZIKV IgG response peaking at day 233, with minor cross-reactions to DENV WV and TBEV WV.

Although ZIKV IgM in most cases was directed against ZIKV NS1, there were IgM responses to YFV NS1 in two patients (Z4, Z57), leading to possible misinterpretation in singleplex assays. Data reduction removed all non-ZIKV reactions in both IgM and IgG. Urea treatment left only specific reactions to ZIKV NS1 for both IgM and IgG.

Case Z1 was a male returning to Germany from Brazil. ZIKV IgM was detected in serum by IIFT on day 10. Figure 1 shows an almost exclusive anti-ZIKV IgM response, peaking at day 20, and a subsequent anti-ZIKV IgG response peaking at day 37, with major probable cross-reactions to DENV WV and to TBEV WV. Both DENV and TBEV IgG reactions were incomplete, i.e., they lacked NS1 reactions which are common in a DENV or a TBEV infection, respectively. The DENV reactions were judged to be cross-reactions from ZIKV. For TBEV there were two possibilities. Either the reaction was due to a cross from ZIKV, or a remnant after TBEV vaccination. Avidities tend to be high after repeated vaccinations (our unpublished observations) while cross-reactions from DENV and ZIKV have low avidities. Occasional avidity values above 1 are likely to be due to a sum of signals from the first incubation without urea and the second one with urea. See also Fig. 6. The data reduction includes these considerations. It removed all DENV reactions but left a significant TBEV IgG score, favoring a past TBE vaccination.

### Flavivirus antibody patterns during DENV infection

Eleven of the 37 DF patients yielded follow-up samples. A DENV response to both WV and NS1 dominated for both IgM and IgG. Minor probable IgM cross-reactions were found to ZIKV E. For IgG, major cross-reactions were shown as TBEV WV and to ZIKV E. DENV antibody kinetics paralleled those of ZIKV and TBEV reactions (data not shown), another sign of cross-reaction from DENV.

**Fig. 3:**
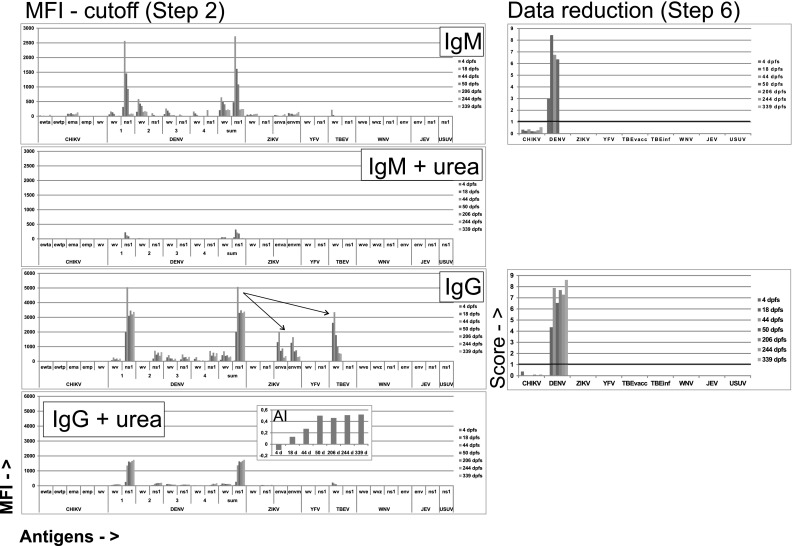
Antibody evolution in a multiply sampled DF case (D51). Avidity index (AI) was calculated for DENV NS1 IgG. See legend of Fig. 1 for further explanations

Case D51 was a male returning to Germany from Sri Lanka. Figure 3 shows an almost exclusive anti-DENV IgM response, peaking at day 18, and a subsequent anti-DENV IgG response peaking at day 44, staying high as long as observed (339 days). The IgG response to DENV NS1 underwent an avidity maturation (insert of Fig. 3). Other DF cases had shorter follow-up times and did not display a clear avidity maturation over time. Possible IgG cross-reactions from DENV occurred on ZIKV E and TBE WV. Both were much reduced by urea treatment (AI 0.15 for ZIKV E antigens and 0.15 for TBEV WV). Both reacting antigens were singular within ZIKV and TBEV, respectively, and therefore likely cross-reactions from DENV. The sample-specific portion of the diagnostic support procedure (“data reduction,” step 6 of Table 1) eliminated both. Although DENV IgM in DF cases was directed against DENV NS1 and/or DENV WV, there were IgM responses to other flavivirus antigens in some DF patients, leading to possible misinterpretation in singleplex assays, to TBE WV (D1, D3, D32, D40, D45, D46, D55, D59), ZIKV NS1 (D56, D68), and to YFV NS1 (D2, D62). Ten of these 12 reactions had an avidity less than 0.2, and were judged as cross-reactions.

### Time course of homologous and heterologous (cross-reactive) flavivirus antibodies during ZIKV and DENV infection

**Fig. 4:**
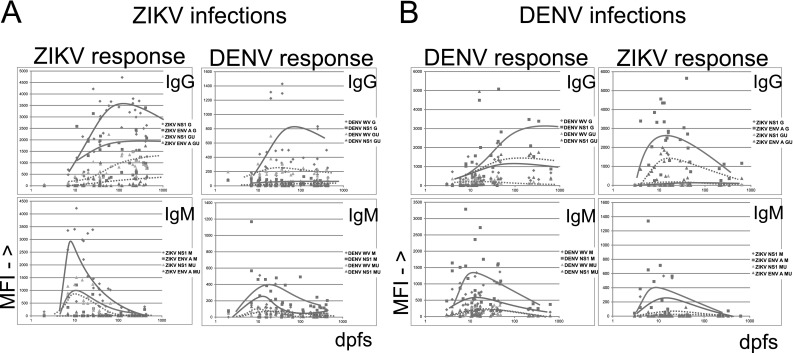
Temporal evolution of homo- and heterologous flavivirus antibodies in 13 ZVD (**a**) and 37 DF (**b**) cases, in sera where the days post first symptom (dpfs; shown in ^10^log form) were known. *Lines* depicting tendencies for IgG and IgM evolution with (GU, MU, *dotted lines*) and without (G, M, *whole lines*) urea treatment are shown. For abbreviations, see legend of Fig. 1

As expected, homologous IgM for both ZVD and DF peaked at 10–20 days post first symptom, while homologous IgG plateaued from about 100 days post first symptom. Heterologous IgM (mainly ZIKV to DENV NS1 and DENV to ZIKV E) did not differ kinetically from homologous IgM. Heterologous IgG had similar kinetics to homologous IgG for ZIKV to DENV WV. In contrast, heterologous IgG to ZIKV E had different kinetics in DF cases. This cross-reaction was most frequent early in infection, peaking at 20–50 days post first symptom (Fig. 4).

### Frequency and magnitude of intra-flavivirus cross-reactions

**Fig. 5:**
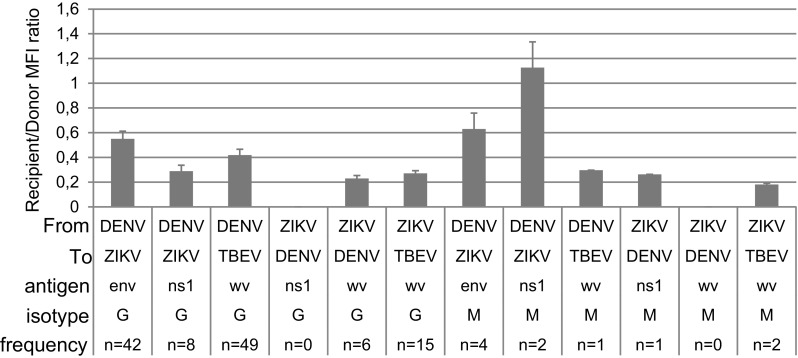
Extent and frequency of probable cross-reactions, represented as the average MFI ratio between heterologous, recipient, flavivirus antigen (”To”), and donor flavivirus (”From”) signal, for DENV WV + NS1 and for ZIKV NS1, respectively. Only cross-reactions of more than 100 MFI were included. *Error bars* denote standard error of mean. See legend of Fig. 1 for further explanations. Results from patients with known YFV and TBEV vaccinations were excluded

We calculated the degree and frequency of cross-reaction to other flaviviruses in PFSMIA by using ZVD and DF samples from patients without known previous YFV vaccination and TBEV infection and vaccination, i.e., 8 ZVD and 35 DF patients. The assumption was that patients were only infected by one virus at a time and previously flavivirus naive. IgG cross-reactions (Fig. 5) from DENV infections were mostly observed as ZIKV E and TBEV WV, and from ZIKV infections to DENV and TBEV WV. IgM cross-reactions were generally weaker and less frequent. They were mainly from DENV to ZIKV NS1, and from ZIKV to DENV NS1. The standard error of the mean was 4–18%, showing a limited variation. The mean of these cross-reactions (“xfactors”) were used to weight possible cross-reactions in the diagnostic support algorithm. A major difficulty is to distinguish TBE vaccination from DENV and ZIKV to TBEV cross-reaction. Both give rise to TBEV WV, but not TBEV NS1 antibodies. Infections tend to give reactions to several, coincident, antigens from the same virus. Avidity offers an additional way to discern homologous antibodies resulting from repeated antigen exposures, like a vaccination, from heterologous ones, arising from a cross-reaction.

### Enhanced specificity of ZIKV and DENV PFSMIA after urea treatment

It is reasonable to assume that cross-reactions, i.e., binding of an antibody elicited by one virus to an antigen from another (heterologous) virus have a lower avidity than homologous antigen–antibody combinations. We therefore investigated whether treatment of beads which already had been measured, with urea, would reveal avidity differences between homologous and heterologous antigen–antibody combinations. This proved to be the case for some combinations. As shown in Fig. 1 for patient Z3, urea treatment brought a general decrease of MFI, but most of the already weak cross-reactions disappeared. Figure 2 (patient Z1), with more of probable cross-reactions, showed persistent TBEV reactions after urea in early sera (consistent with a previous TBEV vaccination), but decrease in cross from ZIKV to DENV WV.

Figure 3 (patient D51) demonstrated that the prominent ZIKV E reactions in this DF case disappeared after urea treatment. The diagnostic support procedure declared these reactions to be cross-reactions, because there were no ZIKV NS1 and WV reactions and they had low avidity. Among all sera, the major cross-reactions were with IgG and occurred between DENV and TBE WV and between DENV and ZIKV E protein (Fig. 5). Urea treatment enhanced the interpretation in some cases. In DF patients, IgG crosses from DENV to TBEV WV decreased in frequency from 46 of 54 DENV positive sera (using a cutoff of 50 MFI) before to 24 of 46 after urea treatment (*p* = 0.0004, Fisher exact test; data not shown). Likewise, IgG crosses from DENV to ZIKV E protein decreased from 45 of 54 before to 19 of 46 (*p* < 0.0001, Fisher) after urea. Other IgG crosses from DENV were not as frequent, and did not decrease significantly after urea treatment. IgM crosses from DENV to ZIKV E in DF patients decreased from 15 of 59 to 2 of 30 (*p* = 0.045, Fisher). Other IgM crosses did not decrease significantly. Thus, urea treatment assisted in the delineation of some, but not all, true (homologous) from false (heterologous) flavivirus antibody reactions. The diagnostic support procedure took advantage of these results (steps 3 and 5, Table 1).

Five chikungunya antigens were included as a check for possible non-specific reactions to a clinically important non-flavivirus. We saw no signs of such cross-reactivity. The antigen panel should be further evaluated with samples from known chikungunya infections.

**Fig. 6:**
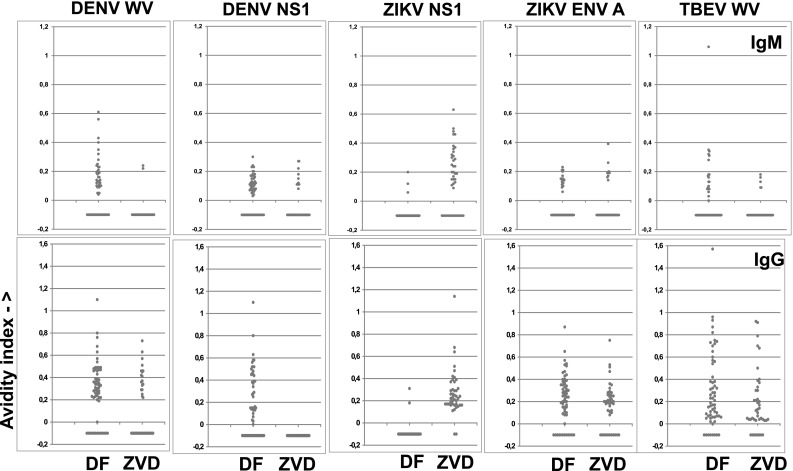
Avidity indices (AI; ratio of MFI after and before urea, *Y*-axis) of the antibody reactions of selected antigens with sera from patients with DF (*n* = 61) and ZVD (*n* = 43). *Upper frames* IgM, *lower frames* IgG. Each *frame* shows the AIs for a DENV, ZIKV, or TBEV antigen, either WV (WV) or NS1. AIs were not calculated if the reactivity of urea untreated bead was less than 300 MFI. Sera with such results are shown as −0.1 on the *Y*-axis. Only results with selected antigens are shown

A more detailed account of the avidities of homo- and heterologous reactions is given in Fig. 6. For IgM, AI of 49 DF sera for DENV WV was 0–0.8, whereas there was a measurable avidity in only two ZVD sera for DENV WV. DENV NS1 avidity did not differentiate DF and ZVD. In contrast, ZIKV NS1 preferentially gave avidities of 0.1–0.6 with 27 ZVD sera but reacted with an avidity of 0.1–0.2 in only three DF sera. Sixteen DF sera gave avidities of 0-0.35 with TBEV WV, while only five ZVD sera gave avidities of 0.1–0.2. For IgG, DENV WV and TBE WV did not yield a differential avidity pattern in DF and ZVD. However, DENV NS1 gave avidities of 0–1.1 in 26 DF sera and none of the ZVD sera. In contrast, 41 ZVD sera gave avidities of 0.1–1.1, whereas three DF sera gave avidities of 0–0.3 with ZIKV NS1. At a cutoff of 300 MFI (necessary for accurate AI calculation), the avidity differences for ZIKV E protein became less marked than at a cutoff of 50 MFI (see discussion of Fig. 6 above). Thus, based on avidity, the antigens which differentiated between ZIKV and DENV most were DENV WV and ZIKV NS1 for IgM, and DENV NS1 and ZIKV NS1 for IgG.

The urea treatment diminished the MFI for homologous reactions to 10–110% of the value for non-treated beads. A dilution of 1/2700 turned out to give approximately the same MFI for ZIKV NS1 as the same serum diluted 1/100 and urea-treated. Urea treatment reduced the heterologous (cross-) reactions relative to the homologous ones (primarily anti-ZIKV NS1) more than the additional dilution (data not shown). We conclude that urea treatment is a more stringent way of selecting for avidity than performing an analysis at a high dilution.

As mentioned, the frequent occurrence of TBEV vaccination in Northern European countries creates a problem of distinguishing false positive (cross) reactions from antibodies elicited by TBEV vaccination. Both yield IgG to WV, but not to TBEV NS1. The judgement of TBEV status, either infection or vaccination, is further detailed in the Supplementary material.

### Final evaluation of PFSMIA results in ZIKV and DENV infection: the diagnostic support procedure

Serological discrimination of ZVD and DF is a major diagnostic problem, and is the main subject of this paper. Table 4 demonstrates the degree of ZIKV/DENV discrimination in four steps of the diagnostic support procedure, leading to incremental accumulation of evidence for a ZIKV and/or DENV infection. The most discriminating situations, which have a sensitivity of over 50%, are shown in bold.

**Table 4:** ZIKV/DENV antibody discrimination in the four main computational steps

Per serum, without and with () urea, MFI-cutoff > 100	ZIKV IgM	ZIKV IgG	DENV IgM	DENV IgG
Step 2 (subtraction of cutoff)				
ZVD (*n* = 43)	NS1: 29(14)	NS1: 41(39)	NS1: 26(5), WV: 9(1)	NS1: 3(1), WV: 33(30)
DF (*n* = 61)	NS1: 3(0)	NS1: 5(1)	NS1: 57(24), WV:54(19)	NS1: 40(32), WV: 57(53)
ZVD/DF discrimination ratio	NS1: 0.67/0.05 = **13.5** (0.33/0) = ∞	NS1: 0.95/0.08 = 11.6 (0.91/0.016 = **55.5**)	NS1: 0.60/0.93 = 0.64 (0.11/0.39 = **0.28**) WV: 0.21/0.89 = **0.24** (0.023/0.31 = 0.07)	NS1: 0.0697/0.656 = **0.11** (0.0232/0.524 = **0.04**) WV: 0.767/0.934 = 0.82 (0.697/0.869 = 0.80)

Per serum, without and with () urea, final score > 1	ZIKV IgM	ZIKV IgG	DENV IgM	DENV IgG
Step 6 (coincidence, average cross-reactivity, avidity)				
ZVD (*n* = 43)	22 (6)	35 (26)	0 (0)	0 (0)
DF (*n* = 61)	1 (0)	0 (0)	39 (3)	36 (26)
ZVD/DF discrimination ratio	0.51/0.016 = **32.0** (0.14/0 = ∞)	0.81/0 = ∞ (0.60/0 = ∞)	0/0.64 = **0** (0/0.05 = 0)	0/0.59 = **0** (0/0.43 = 0)

Per patient, without urea, at least twofold increase (!) or decline (§)	ZIKV NS1 IgM	ZIKV NS1 IgG	DENV NS1 or WV IgM	DENV NS1 or WV IgG
Step 7 (reactivity change)				
ZVD, *n* = 13, of which 11 were multiply sampled, (nr with change)	!2 §10 (10)	!7 §2 (9)	!0 §0 (0)	!0 §0 (0)
DF, *n* = 37, of which 12 were multiply sampled, (nr with change)	!0 §0 (0)	!0 §0 (0)	!2 §5 (7)	!8 §1 (9)
ZVD/DF discrimination ratio	**0.91**/0 = ∞	**0.82**/0 = ∞	0/**0.58** = **0**	0/**0.75** = **0**

Per patient	Primary ZVD	Previous ZVD	Primary DF	Previous DF
Step 8 (compound judgment)				
ZVD (*n* = 13)	12	1	0	0
DF (*n* = 37)	0	0	35	2
ZVD/DF discrimination ratio	1/0 = ∞		0/1 = **0**	

Numbers shown in bold emphasize a major discrimination of ZVD and DF

After cutoff subtraction (step 2), ZIKV NS1 IgM without urea, and ZIKV NS1 IgG with and without urea were highly discriminatory (ratios of 13.5, 1.6, and 55.5, respectively), yet sensitive (65, 95, and 91%, respectively) for ZVD.

Regarding DF, DENV WV IgM without urea together with DENV NS1 IgM with urea and for IgG DENV NS1 IgG without and with urea, were specific for DF (ZVD/DF ratios of 0.24, 0.28, 0.11, and 0.04, respectively). These combinations were also relatively sensitive for DF (89, 39, 66, and 52%, respectively). Thus, a combination of WV and NS1 was necessary for DENV antibody detection. Reliance on one variable was not enough.

After calculation of final score per serum, based on coincidence, average cross-reactivity and avidity (step 6), gave a high specificity for ZIKV IgM without urea. Fifty-one percent of ZVD sera became positive, confirming that IgM alone could not detect all ZVD. ZIKV IgG was more sensitive (81% of ZVD detected), yet specific (no DF positive). DENV IgM without urea detected 64% of DF sera, with high specificity. Likewise, DENV IgG without urea detected 59% of DF sera, with high specificity.

Detection of reactivity changes per multiply sampled patient (step 7), gave a high sensitivity for ZIKV, (91%, IgM, 82%, IgG) and specificity (0% false positive for IgM and IgG). Likewise, sensitivity for DENV changes was relatively high (58%, IgM, 75%, IgG).

Incorporating information from the previous steps, plus gathering of evidence for a primary or previous ZIKV or DENV infection (step 8), sensitivity and specificity became 100%, for both ZDV and DF, under the chosen conditions.

As seen in Tables 3 and 4, urea treatment weakened reactions and sensitivity significantly, especially for IgM, but increased specificity, especially for ZIKV and DENV NS1 IgG. The ability to detect reactivity changes for ZDV/DF discrimination was highest without urea treatment. A diagnostically useful change, twofold or higher, was generally a decline for IgM and an increase for IgG. The most diagnostically useful and common reactivity changes (Table 1 step 7) were IgM declines in ZVD and IgG increases in DF cases.

Results per serum and with activity changes, are collectively shown in Table 3 as “Main serological reactions” (steps 6 and 7 of Table 1) and per patient as “Case judgement” (step 8 of Table 1). The column “Main serological reactions” contains a few additional reactions which are not congruent with ZVD and DF. YFV and TBEV reactions may have been due to undocumented vaccinations. Others, like two reactions to USUV ns1, are hard to corroborate, and should be investigated. The degree of diagnostic concordance is shown in column “Concordance” of Table 3. An “OK” in this column indicates that the diagnostic support procedure gave the same result as other methods (“Previous diagnostic evidence”). An “OK?” indicates a slight deviation versus other methods, or unknown status (YFV in case D2, ZIKV in case D56, and YFV? in case D62). 48 of 50 cases got an “OK,” 2 got an “OK?” None gave an erroneous result.

There were a few problems when trying to interpret the PFSMIA patterns in a broader context than ZDV/DF discrimination. For example, we did not have a complete vaccination record, or a record of previous flavivirus infections. The interpretation of previous flavivirus infections (based on IgG) was therefore tentative. As seen in Table 3, serum from D69 gave a relatively weak IgM DENV reaction, and was reported with a question mark. Z54 and D2 had IgM reactive to YFV NS1, indicating a recent YFV infection or vaccination. Patients D56 and D68 reacted strongly by IgM to DENV and weakly with several ZIKV antigens in early samples. Subsequent samples were ZIKV negative in D56. This seems to be a rare IgM cross-reaction from DENV to both ZIKV NS1 and ZIKV E that could not be compensated for using the coincidence criterion.

### Applying PFSMIA to sera from blood donors and pregnant women

**Fig. 7:**
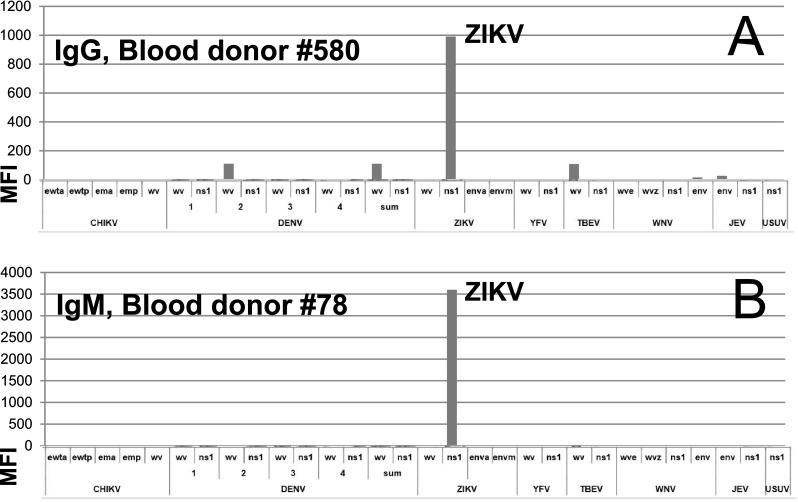
Blood donor sera which reacted strongly with ZIKV NS1.** a** A blood donor serum reactive in IgG.** b** A blood donor serum reactive in IgM. For abbreviations, see the legend of Fig. 1

None of 200 sera from pregnant women reacted with ZIKV NS1. Two of 173 blood donor sera had strong and exclusive ZIKV NS1 reactivities. The PFSMIA profiles of the ZIKV NS1 antibody positive donors #580 (IgG) and #78 (IgM) are shown in Fig. 7. The NS1 reactions were at least as strong as those of confirmed Zika IgM and IgG positive cases, respectively. The absence of reactions to the other ZIKV antigens (WV and E) argues for the results to be non-specific. However, the IgG AI of #580 for ZIKV NS1 was 0.21. IgM AI of #78 for ZIKV NS1 was 0.18. Both AIs were thus measurable but not strongly advocating a homologous reaction. We therefore further evaluated blood donor sera #580 and #78 in a commercial Zika NS1 EIA (Euroimmun AG). With a cutoff of OD 1.1, the former became borderline IgG positive (OD 1.0), while the latter reacted in IgM (OD 1.4). We also tested the sera in IIFT using ZIKV-infected cells. Serum #78 gave an ambiguous IgM reactivity up to 1/40, while #580 gave an ambiguous IgG reactivity up to 1/20. Serum #580 was negative in ZIKV IgM IFA, EIA, and SMIA, and #78 for ZIKV IgG IFA, EIA, and SMIA. Serum #78 was negative in a sensitive ZIKV RT-PCR. One blood donor and 7 pregnant women reacted in IgG, but not in IgM, with DENV WV and/or DENV NS1.

## Discussion

Flaviviruses can emerge rapidly [19–22], emphasizing the need for broad surveillance. Aside from YFV and TBEV vaccination, the European population is largely flavivirus naive, which somewhat simplifies serological testing. Several factors contributed to give a high analytical specificity of PFSMIA; a low background for most sera, a high precision and simultaneous measurement of all flavivirus antibodies in the same reaction. Although single antigen EIAs are cumbersome this limitation has been addressed [23]. Multiplex flavivirus serology in a more limited form has recently been reported by others [24]. The PFSMIA intra-flaviviral cross-reactions described here agree with observations obtained by other techniques [3–5, 8, 9]. Antibody cross-reactions occur between the various flaviviruses, and also with host proteins [25, 26]. Our aim was to distinguish between true and false (cross-) reactions, either numerically, using isotype specific cross-reaction factors and coincidence criteria, or based on avidity, using a second run after urea treatment. The extent of cross-reactions differed for WV, E protein, NS1 antigens, and also for the Ig type (IgG or IgM), enabling a tailor-made computational correction. ZIKV IgM gave the least ambiguous results, i.e., the highest specificity. Most heterologous reactions had approximately the same kinetics, studied at different times post first symptom, as homologous ones. However, heterologous IgG to ZIKV E was most frequent early in DF. The compensation assumes a relatively constant degree of cross-reactivity for each heterologous combination. Although the variation of the most frequent cross-reactions was limited in this study, this may not always be the case. However, the compensation mechanism correctly classified cases using the 104 sera analyzed here. ZIKV and DENV NS1 IgM had a smaller tendency for cross-reaction than NS1 IgG. However, NS1 IgM assays are not immune to error. For example, a deficient specificity was shown by the singular ZIKV NS1 IgM reaction of blood donor #78, and a deficient sensitivity by DENV NS1 IgM alone in DF. Thus, both DENV WV and NS1 were required, and multiplexity combined with avidity determination was proven superior to singleplex NS1 assays.

In the studied North European population, the major confounding factor for flavivirus serology is TBEV vaccination. Numeric cross-reactivity compensation was concordant with avidity in cases with TBEV WV but not NS1 IgG. In populations with a higher frequency of flavivirus infections than in Europe, it can become increasingly difficult to distinguish the infection history due to enhanced cross-reactions arising from anamnestic reactions (i.e., “original antigenic sin”). This phenomenon could be the reason for the reverse DENV IgG avidity evolution in the ZIKV-infected patient Z1. We postulate that IgM and IgG memory cells [27] remaining from a previous exposure to another flavivirus, in this case DENV and/or TBEV, are quickly activated to produce DENV- or TBEV-specific immunoglobulin, respectively, then gradually undergoing avidity maturation towards ZIKV, the emerging clones becoming less specific and less avid to their original targets. Such phenomena can complicate serological interpretation, both by human and computer.

The use of avidity selection for studying the maturation of antibody responses is well established in clinical virology [28, 29]. We here demonstrate that it also can be an aid for distinguishing specific (homologous) flavivirus antibody reactions from non-specific (heterologous) ones. The observed specificity-enhancing effect was clearer for ZIKV than for DENV infections. A complicating factor regarding DENV is that there are four serotypes, increasing the chance for a non-perfect fit between patient antibody and PFSMIA antigen, resulting in lower avidity in antibody–antigen combinations. Avidity may become useful for seroepidemiological studies and determination of vaccination status. Our rational procedure is based on the re-use of magnetic beads and antigen–antibody complexes.

Judging from the 104 sera tested here, the diagnostic support procedure increases the specificity incrementally, without a significant decrease in sensitivity. Step 2 eliminates most antibody reactivity not directed to flaviviruses. Steps 3–5 (avidity, coincidence, xfactors) lead to calculation of final score per serum (step 6). Most cross-reactions (e.g., DENV to ZIKV env; TBEV to DENV WV; DENV to TBEV WV) are eliminated here. Reactivity rise and decrease (step 7) is a time-honored clinical virological diagnostic modality [30]. Significant reactivity changes occurred both for IgM and IgG, and added specificity to the diagnostic judgements. Reactivity changes and antibody isotype (IgM/IgG) were weighed together in step 8, to provide a judgement on ZIKV or DENV infections of the patient (the case). These four layers of computation contributed to the diagnostic specificity. The procedure is automated, and can be integrated into a high-throughput serological screening system. Further details are given in [16].

In rare cases, ZIKV has been transmitted via blood transfusion, and testing of blood donors or pregnant women is at present discussed [31–34]. Although there are guidelines [35], the exact criteria for ZIKV seropositivity in a screening situation are still not well defined. NS1 is highly antigenic and the most specific ZIKV antigen. DENV NS1 cross-reacts with host proteins [26]. The extent of such cross-reactions for ZIKV NS1 is still unknown, but may explain the observed blood donor reactions (which were confirmed in commercial singleplex EIAs). An alternative explanation, that ZIKV infection occasionally occurs among Swedish blood donors, would be both surprising and alarming. Isolated NS1 antibody reactions can give problems if ZIKV antibody screening is contemplated. Such blood donor samples should be thoroughly investigated.

Although here evaluated by ZIKV- and DENV-specific sera, the techniques described here seem to have the potential to provide a comprehensive flavivirus antibody classification. A future larger study is needed to establish this in detail.

Diagnosis of ZIKV and other flavivirus infections requires several independent analytical modalities. PCR, NS1 antigen tests, virus isolation, and comparative neutralization tests, preferably in a rational format [36], can be used for supplementation of PFSMIA results when a case requires a thorough investigation. The limited time span of viremia necessitates, however, an optimal serology. Our novel computerized and avidity-based interpretation of heterologous from homologous IgM and IgG reactions from results with both single and multiple sera from the same patient described here provides a comprehensive, yet high-throughput, support for efficient flavivirus antibody-based diagnosis. It is simple to perform, and may diminish the need for additional labor and time-consuming tests.

## Electronic supplementary material

Below is the link to the electronic supplementary material.

Supplementary material 1 (DOCX 27 kb)
