# Supplementary material for: Compensating for cross-reactions using avidity and computation in a suspension multiplex immunoassay for serotyping of Zika versus other flavivirus infections
Source: Med Microbiol Immunol. 2017 Aug 29;206(5):383–401. doi: 10.1007/s00430-017-0517-y (PMC5599479; doi:10.1007/s00430-017-0517-y)
Supplement: Supplementary file 1 — Supplementary material 1 (DOCX 27 kb) [file 430_2017_517_MOESM1_ESM.docx]

Supplementary material, “**Compensating for cross-reactions using avidity and computation in a suspension multiplex immunoassay for serotyping of Zika and other flavivirus infections”**

**Table S1.** Calculation of cutoff for each antigen. For abbreviations and antigen details, see Materials and Methods.

| **Virus** | **Antigen** | **Cutoff for IgG**  **(Average+3SD, MFI)** | **Sero-negative**  **Blood donors (BD), average**  **IgG** | **Sero-negative**  **BD, SD,**  **IgG** | **Cutoff for IgM,**  **(Average**  **+3SD, MFI)** | **Sero-negative**  **BD average,**  **IgM** | **Sero-negative**  **BD, SD,**  **IgM** |
| --- | --- | --- | --- | --- | --- | --- | --- |
| CHIKV | wild type E1, Aalto | 370 | 85 | 95 | 339 | 85 | 85 |
| CHIKV | wild type E1, Prospec | 607 | 84 | 174 | 605 | 155 | 150 |
| CHIKV | mutant E2, Aalto | 390 | 86 | 102 | 233 | 64 | 56 |
| CHIKV | mutant E2, Prospec | 20 | 2 | 6 | 169 | 39 | 43 |
| CHIKV | WV | 8 | 1 | 3 | 3 | 0 | 1 |
| DENV 1 | WV | 42 | 6 | 12 | 79 | 22 | 19 |
| DENV 1 | NS1 | 21 | 3 | 6 | 235 | 62 | 58 |
| DENV 2 | WV | 650 | 290 | 120 | 85 | 30 | 19 |
| DENV 2 | NS1 | 16 | 1 | 5 | 55 | 10 | 15 |
| DENV 3 | WV | 61 | 15 | 15 | 58 | 24 | 11 |
| DENV 3 | NS1 | 35 | 3 | 11 | 61 | 11 | 10 |
| DENV 4 | WV | 149 | 63 | 29 | 67 | 15 | 18 |
| DENV 4 | NS1 | 134 | 22 | 37 | 217 | 52 | 55 |
| ZIKV | WV | 174 | 99 | 25 | 92 | 21 | 23 |
| ZIKV | NS1 | 25 | 1 | 8 | 317 | 61 | 85 |
| ZIKV | E Aalto | 1013 | 308 | 235 | 307 | 104 | 68 |
| ZIKV | E Meridian | 500 | 185 | 180 | 221 | 72 | 50 |
| YFV | WV | 108 | 42 | 22 | 16 | 3 | 5 |

**Table S2.** Flavivirus antibody reference sera and their PFSMIA results.

| **Sample** | **Serum catalog nr** | **Dilution** | **Result with other test** | **Classification, PFSMIA**  **Diagnostic support procedure,** |
| --- | --- | --- | --- | --- |
| West Nile Virus IgG Pos | ZeptoMetrix Serodetect WNV1 KZMC027, lot 1301-272-00150 | 1/80 | Focus WNV IgG EIA 4.73 | WNV |
| West Nile Virus IgG Pos | ZeptoMetrix Serodetect WNV3 KZMC027, lot 1301-272-00150 | 1/80 | Focus WNV IgG EIA 5.19 | WNV |
| West Nile Virus IgG Pos | ZeptoMetrix Serodetect WNV4 KZMC027, lot 1301-272-00150 | 1/80 | Focus WNV IgG EIA 5.26 | WNV |
| West Nile Virus IgG Pos | ZeptoMetrix Serodetect WNV6 KZMC027, lot 1301-272-00150 | 1/80 | Focus WNV IgG EIA 5.29 | WNV |
| West Nile Virus IgG Pos | ZeptoMetrix Serodetect WNV7 KZMC027, lot 1301-272-00150 | 1/80 | Focus WNV IgG EIA 4.45 | WNV |
| West Nile Virus IgG Pos | ZeptoMetrix Serodetect WNV10 KZMC027, lot 1301-272-00150 | 1/80 | Focus WNV IgG EIA 5.40 | WNV |
| WHO Yellow fever IgG Pos | NIBSC YF | 1/160 | Krag et al Bull Wld Hlth  Org  1965, 33:243-245 | YFV |
| WHO Louping Ill (Moredun) Sheep IgG Pos | NIBSC TILI | 1/80 | - | TBEinf |
| WHO Tick Borne Encephalitis (Sophyn and Absettarov) Sheep IgG Pos | NIBSC TISA | 1/80 | NT test | TBEinf |

**Result details which could not be included in the main text.**

Judgement of serological TBEV_status

After the statistically based subtraction of TBEV signal (cf. Figure 2) in the DF and ZDV cases the algorithm (step 6, Table 1) predicted that 13 of 36 cases with TBEV WV IgG and no TBEV NS1 IgG were due to vaccination, while 23 were due to cross-reaction. The average TBV WV avidity of sera from the 13 alleged vaccine reactions was 0.5 (SD 0.27), while the 23 alleged cross-reactions had an average of 0.06 (SD 0.14). Regarding concordance with known vaccinations, TBEV vaccinated patients were reported as TBEV vaccinated with or without a question mark in 3 of 5 known cases. One (Z6) reacted with TBEV WV, but not strong enough to be reported as vaccinated here. Another (D62) reacted both with WV and NS1, indicating a past TBE infection. Z56 reacted weakly in PFSMIA with YFV, not strongly enough for mentioning in the case judgement. Thus, although the concordance was not perfect, there was a serological reaction corresponding to the known vaccination in all cases. A larger evaluation of TBEV vaccination and infection testing by PFSMIA is in preparation.

Correlation of antibody reactivity to DENV type antigens with known DENV type

An imperfect correlation between DENV type and maximally PFSMIA-reactive DENV type WV and NS1 antigen was seen (data not shown). The highest correlations with DENV type (determined with PCR and DENV NS1 antigen) were obtained with sera with a strong reaction with both WV and NS1 for the DENV type. Two thirds (66%) of the cases reacted in the expected fashion, the rest did not (data not shown). The typing ability was not improved after urea treatment.
